# The evidence and impact of deprescribing on sarcopenia parameters: a systematic review

**DOI:** 10.1186/s12877-025-05819-7

**Published:** 2025-03-07

**Authors:** Kinda Ibrahim, Natalie J. Cox, Stephen E. R. Lim, Eloise Radcliffe, Carina Lundby, Konstantinos Prokopidis, Wade Thompson, Frank Moriarty

**Affiliations:** 1 School of Primary Care, Population Sciences and Medical Education, Primary Care Research Centre and the NIHR Applied Research Collaboration (ARC) Wessex, University of Southampton, Southampton, UK; 2 Academic Geriatric Medicine, Human Development and Health, Faculty of Medicine, NIHR Applied Research Collaboration (ARC) Wessex, University of Southampton, Southampton, UK; 3 Clinical Pharmacology, Pharmacy and Environmental Medicine, Department of Public Health, University of Southern Denmark and Hospital Pharmacy Funen, Odense University Hospital, Odense, Denmark; 4 Department of Musculoskeletal Ageing and Science, Institute of Life Course and Medical Sciences, University of Liverpool, Liverpool, UK; 5 Department of Anaesthesiology, Pharmacology, and Therapeutics, Faculty of Medicine, University of British Columbia, Vancouver, Canada; 6 School of Pharmacy and Biomolecular Sciences, RCSI University of Medicine and Health Sciences, Dublin, Ireland

**Keywords:** Sarcopenia, Deprescribing, Muscle strength, Muscle mass, Muscle function, Outcomes

## Abstract

**Background:**

Polypharmacy (concomitant prescription of ≥ 5 medications) affects a third of older people, and evidence suggests an association with sarcopenia (loss of skeletal muscle mass/quality, muscle strength, and/or physical performance). As such, deprescribing has been recommended in routine management of sarcopenia, however it’s unknown whether deprescribing is beneficial. This systematic review aimed to understand effects of deprescribing on sarcopenia parameters in older adults.

**Methods:**

Medline, Embase, CINAHL, Web of Science, and the Cochrane Library databases were searched up to July 2023. All studies reporting effects of deprescribing interventions on sarcopenia parameters (primary outcomes) or nutritional intake (secondary outcomes) among older adults were included. Findings were summarised narratively, and study quality was assessed.

**Results:**

A total of 4860 articles were identified and six were included (mean age range 67–87 years). Studies were heterogeneous in design, settings, follow-up periods, and outcomes. Deprescribing had no effect on skeletal muscle mass (*n* = 2). Positive effects were shown on handgrip strength with two studies reporting improvements following antihypertensive or benzodiazepines discontinuation and one showing no change between admission and discharge with general deprescribing. Outcomes of deprescribing on physical function outcomes varied based on the measures used. For example, one study showed no changes in timed up and go, Whereas effects on gait speed was contradictory in two studies, with preservation and deterioration reported. Two studies reported improvement between baseline and follow up in balance scores measured part of the Short physical performance battery or using the Short Berg’s Balance Scale among those who discontinued antihypertensive and/or benzodiazepines. Two studies reported improvements in nutritional outcomes following deprescribing at hospital discharge, whereas two other studies reported no change or increase in weight loss.

**Conclusion:**

There is limited research about the impact of deprescribing on sarcopenia parameters. This systematic review found no significant changes in muscle mass but there is some evidence in improvements in strength, physical performance, and nutritional status with deprescribing. The multidisciplinary implementation of nutrition and exercise therapies, as well as medication management to modify polypharmacy, may further promote improvement in sarcopenia. However, more high-quality research is needed to understand the effects of deprescribing on sarcopenia parameters among older people including those with confirmed diagnosis of sarcopenia.

**Registration:**

The review was registered on the international prospective register of systematic reviews (PROSPERO, CRD42023417997).

**Supplementary Information:**

The online version contains supplementary material available at 10.1186/s12877-025-05819-7.

## Background

One-third of people aged 65 years and over live with multimorbidity and take five or more regular medicines (polypharmacy), increasing to 50% in those aged 85 years and over [1, 2]. There is increasing interest in understanding the association between polypharmacy and sarcopenia, i.e., loss of skeletal muscle mass or quality, muscle strength, and/or physical performance [1], which affects up to 27% of community-dwelling older people worldwide [2, 3]. Both polypharmacy and sarcopenia are associated with increased risk of falls, cognitive impairment, functional decline, hospitalisation, length of stay, and death [1, 4–7], resulting in an estimated annual excess cost of £2.5 billion in the UK as an example [8].

The European Working Group on Sarcopenia in Older People II criteria (EWGSOP II) revised the definition of sarcopenia diagnosis in 2019 and recommend an updated screening and assessment pathway that is easy to use in clinical practice. The new criteria focus on low muscle strength as a key characteristic of sarcopenia because it is easy to measure in clinical practice and recognised to be better than muscle mass in predicting adverse outcomes. Sarcopenia is probable when low muscle strength is detected, and diagnosis is confirmed by the presence of low muscle quantity or quality. When low muscle strength, low muscle quantity/quality and low physical performance are all detected, sarcopenia is considered severe. The new diagnostic criteria suggest that muscle mass and muscle quality are technically difficult to measure accurately and are used mainly in research rather than in clinical practice.

A 2023 systematic review and meta-analysis identified an association between polypharmacy and sarcopenia or risk for sarcopenia, which might be mediated by other conditions, in particular malnutrition [9]. The causality of this relationship is still undetermined. The physiological mechanisms linking polypharmacy and muscle health are complex and multifactorial. Some drugs used for therapeutic or preventive purposes act on skeletal muscle, and could induce sarcopenia [10]. Drug-related sarcopenia has not yet received much attention. Polypharmacy has been shown to be linked to muscle weakness, poorer physical performance (walking speed, handgrip strength, time to functional recovery), and low appendicular lean mass in older age [11, 12]. More specifically, chronic use of psychotropic medications is associated with functional decline [13]; cardiovascular medications associated with reduced handgrip strength [14]; antipsychotics can cause tardive dyskinesia, a condition involving involuntary muscle movements [15]. Statins may increase the production of reactive oxygen species in muscle tissue contributing to muscle pain and weakness [16]. Chronic use of corticosteroids can result in muscle atrophy and weakness due to their catabolic effects on muscle protein metabolism [17]. Diuretics can cause imbalances in electrolytes (low potassium) which are critical for normal muscle function leading to muscle weakness and cramps and research suggest its associated with higher risk of sarcopenia [18].

Sarcopenia is considered a reversible condition and current guidelines recommend a combination of progressive strength resistance exercise with high-protein diet or nutritional supplementation for management [19]. Some drugs such as fall-risk increasing drugs, psychotropic and anticholinergic medications are shown to impair exercise engagement via their effects on cognition, sedation, orthostasis, or mobility [20]. Additionally, there is an association between polypharmacy and malnutrition (key contributor to sarcopenia) [21, 22] due to potential changes in gastrointestinal microenvironment and the gut microbiota [23]. Therefore, deprescribing (planned, supervised process of reducing or stopping medications) to manage polypharmacy has been suggested for inclusion in routine management of sarcopenia [24, 25]. It has been argued that reducing polypharmacy and prescribing of medications that contribute to muscle wasting and functional decline could improve sarcopenia parameters. However, research on the effects of deprescribing on sarcopenia parameters or factors associated with sarcopenia is scarce and more evidence is needed to inform guidelines for management of sarcopenia [26, 27]. Therefore, the aim of this review was to summarise the effects of deprescribing on sarcopenia parameters (muscle strength, muscle function, muscle mass) in older people who might have sarcopenia.

### Methods

We conducted a systematic review, reported according to the Preferred Reporting Items for Systematic reviews and Meta-Analyses (PRISMA) statement [28]. The review was registered on the international prospective register of systematic reviews (PROSPERO; CRD42023417997) and no changes to the protocol were made.

#### Data sources and searches

The search strategy (supplementary file S1) was developed with a senior librarian using the following databases: Medline, Embase, CINAHL, Web of Science, and the Cochrane Library and was conducted in July 2023. Reference lists of included articles were searched for additional relevant studies. The PICO statements outlined in Table 1 were used to guide the inclusion and exclusion of papers.

**Table 1:** Study inclusion and exclusion criteria

	Inclusion criteria	Exclusion criteria
Population	• Participants average age 65 years and over with or without sarcopenia (based on inclusion/exclusion criteria)	• Participants average age under 65 years
Intervention	• Interventions conducted in any settings including: • Observational studies • Before and after studies • Randomised trials • Non-randomised controlled trials • Interrupted time series • Case studies	• Qualitative studies • Systematic reviews • Process evaluation
	• Intervention focused on deprescribing (i.e., the process of tapering /dose reduction, stopping, or switching drugs, with the goal of improving outcomes) including pure deprescribing interventions or studies reporting deprescribing as part of medication review intervention.	• Intervention for medication review without a clear deprescribing element. • Deprescribing as part of multi-component interventions and outcomes cannot be assigned to deprescribing alone
Comparator	• Any comparator	• N/A
Outcome	• Primary outcome: • Sarcopenia parameters including muscle strength, muscle mass and physical performance • Diagnostic criteria for sarcopenia (i.e., EWGSOP 1 and 2, AWGS, FNIH)	• Studies that do not include outcomes on sarcopenia parameters, or nutrition status/intake
	• Secondary outcomes include nutrition status/intake via any valid screening or assessment method.	

#### Type of studies

We anticipated a small number of studies to explore the effects of deprescribing on sarcopenia parameters among older people. Therefore, observational studies, before and after studies, randomised controlled trials (RCTs), non-randomised controlled trials, interrupted time series, or case studies in any setting were included. Studies involving deprescribing as the only intervention or as part of medication review interventions were included [29].

#### Type of participants

We included deprescribing studies that targeted older people aged 65 years and over.

#### Type of outcomes

We included studies reporting on any of the following primary outcomes for sarcopenia parameters following the EWGOSP II guidelines:

- Muscle strength (for example using grip strength [30] or chair-stand test [31]).
- Physical performance (for example using 4-metre gait speed test [32], Short physical performance battery (SPPB) including balance measures [33], Timed-up-and-go (TUG) test [34], 400-meter walk [35]).
- Skeletal muscle Mass (SMM) or skeletal muscle quality (for example using Bioelectrical impedance analysis (BIA) or Dual-energy X-ray) [36].

Nutrition was chosen as a secondary outcome due to the significant link between malnutrition and sarcopenia from one hand and the association between polypharmacy and nutrition on the other hand, as well as the inclusion of nutrition in the management of sarcopenia guidelines [37]. Nutrition status via any screening or assessment method, for example, total food intake, protein intake, energy intake, or measurement of malnutrition-risk were included.

### Study selection

Each title and abstract of identified articles were independently screened by two authors (KI, SL, ER, WT, CL, FM, KP) using the Rayyan electronic platform to identify studies that met inclusion criteria [38]. Then each full text article identified for possible inclusion was independently reviewed by two authors (KI, NC, WT, KP) to identify relevant studies for final analysis. The references of included articles were also screened for relevance. Following each stage, any disagreement was resolved by discussion among at least two authors.

### Quality assessment

Study quality was assessed separately by two authors (FM & KP) using the standardized Joanna Briggs Institute checklists for each study type, with total scores of 13 for RCTs and 11 for observational studies. Final scoring was agreed by discussion. A score ≥ 7/13 for RCTs and ≥ 5/11 for observational studies were considered to represent good quality [29].

### Data abstraction and synthesis

It was anticipated that there would be substantial heterogeneity in study design and outcome reported, therefore we anticipated that a meta-analysis would not be possible. Hence, a narrative synthesis of the findings was conducted following the Synthesis Without Meta-analysis (SWiM) guideline [39]. Data from included studies were extracted into a pre-defined template for conceptualisation and construction of the systematic review. Data extracted included: year of publication, country, setting, number and age of participants, description of the intervention and any comparator, follow up period, and primary and secondary outcomes reported. Studies were grouped according to the participant groups recruited. Outcome data were summarised for each study and compared.

## Results

**Fig. 1:**
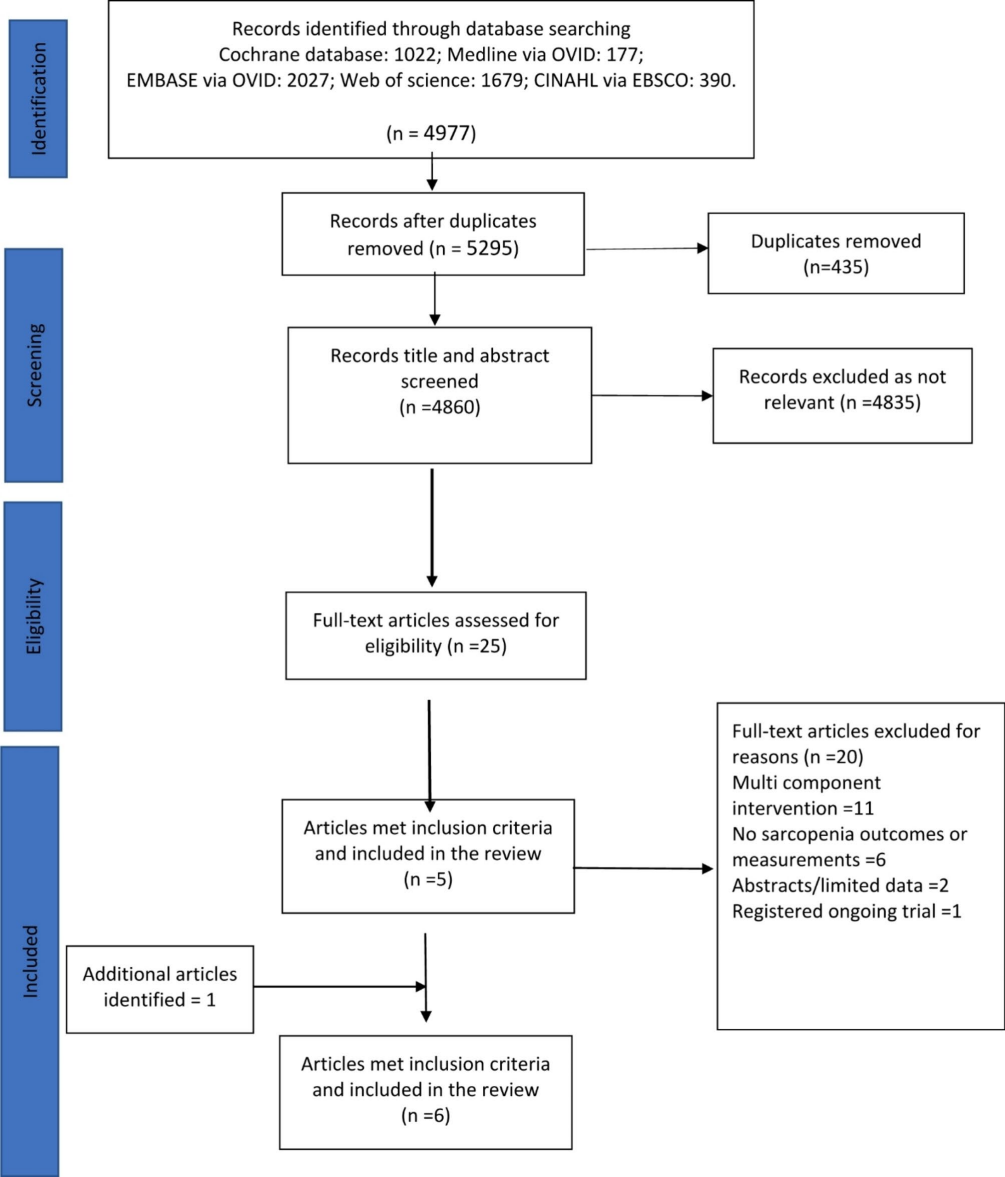
PRISMA flow diagram of study identification and inclusion

Overall, 4860 articles were identified, and 25 articles were selected for full text assessment with five papers met the inclusion criteria. One additional article was identified from the reference lists resulting in six articles included in the review (Fig. 1). The six eligible articles (full study details are presented in Table 2) included five observational studies (one in Turkey [40], one in Finland [41], and three in Japan [42–44]) and one RCT (Australia) [45]. One study was conducted in aged-residential care [45], one in primary care [41], two in outpatient clinics [40, 42], and two of the articles reported separate analyses drawn from the same population in a rehabilitation ward setting [43, 44]. The mean participant age ranged from 67 to 87 years and follow up periods were 107 days [43, 44], 6 months [40–42], and 1 year [45]. The quality of the included articles ranged between 4/11 and 9/11 for the five observational studies and 11/13 for the RCT.

**Table 2:** Details of included studies

Authors (year of publication	Study design and aims	Setting and country	*N* of Participants (age and gender)	Description of the intervention	Comparator (if any)	Study Follow up	Outcome measures	Quality scoring
Janne Nurminen et al. (2014)	Prospective study that aimed to assess outcomes of withdrawal of benzodiazepine and z-drugs on grip strength and balance	Primary care, Finland	89 patients mean age 67) Mean number of medications at baseline = 4	Individual physician-directed withdrawal was performed gradually over a one-month period	short-term withdrawers (1 month) and short-term non-withdrawers long-term withdrawers (> 6 months) and long-term non-withdrawers	6 months	- Handgrip strength - Balance using the Short Berg’s Balance Scale (SPBB-9)	7/11
Eral Idil et al. (2021)	Retrospective and longitudinal study examining the effect of change in drug number on functionality and physical performance.	Outpatient, Turkey	515 participants (age 74 ± 7 years). The baseline mean number of drugs was 5.11 ± 3.34,	Medication review led by geriatricians following the principles of comprehensive geriatric assessment (CGA) and 2015 Beers’ criteria	Baseline compared to 6 month follow up in the deprescribing group (*n* = 219)	6 months	- Gait speed - Timed-Up-And-Go (TUG) Mini nutritional assessment	5/11
Elizabeth E Roughead et al. (2022)	Randomized controlled trial which aims to assess the effectiveness of a pharmacist-led intervention using validated tools to reduce medicine-induced deterioration and adverse reactions.	Aged-care residency, Australia	248 Residents (median age 87years) taking ≥ 4 medicines or ≥ 1anticholinergic or sedative medicine were included from 39 aged-care facilities	Pharmacist-led medication review intervention using validated tools to detect signs and symptoms of medicine-induced deterioration which occurred every 8 weeks over 12 months.	Usual care (Resident Medication Management Review, RMMR) provided every 12–24 months for residents. (9 participants in the control received RMMR)	12 months	- Handgrip strength - Nutrition (weight)	11/13
Sho Hasegawa et al. (2022)	Retrospective chart-review pilot study	Outpatient frailty clinic, Japan	78 patients (discontinuation group, *n* = 19; continuation group, *n* = 59). Median age of 77.0 years (72–82).	Discontinuation or continuation of antihypertensive drugs	Comparison of outcomes between baseline and 1 year follow up visits among two groups discontinuation or continuation of antihypertensive drugs	12 months	- Skeletal Muscle Mass Balance using the Short Physical Performance Battery (SPBB)	4/11
Ayaka Matsumoto et al. (2022)	Retrospective cohort study to investigate the effect of deprescribing for polypharmacy on the improvement of nutritional intake and sarcopenia in older patients with sarcopenia.	Hospital rehabilitation wards, Japan	91 (mean age 81 years) presented with sarcopenia and polypharmacy (6 or more drugs). The median number of medications prescribed at the time of admission was 8 (6–9).	Potentially inappropriate medications (PIMs) were identified by pharmacist according to 2019 Beers Criteria	Admission to discharge across two groups (deprescribing vs. non-deprescribing)	107 days	- Handgrip strength - Skeletal Muscle Mass - Nutrition (energy and protein intake)	9/11
Eiji Kose et al. (2023)	Retrospective cohort study to assess the association between deprescribing and functional recovery and home discharge in older patients with sarcopenia after stroke.	Hospital rehabilitation wards, Japan	153 patients (mean age = 81) were diagnosed with sarcopenia. The median number of medications taken on admission was 7 (range, 5–9).	Potentially inappropriate medications (PIMs) were identified by pharmacist according to 2019 Beers Criteria	Admission to discharge across two groups (deprescribing vs. non-deprescribing)	107 days	- Physical function	9/11

### Presence of sarcopenia

Two of the included articles reported on separate retrospective analyses drawn from the same population of patients with confirmed diagnosis of sarcopenia on a stroke rehabilitation ward [43, 44]. Sarcopenia diagnosis was based on the Asian working group for sarcopenia cut offs for handgrip strength and skeletal muscle mass index. As sarcopenia can be difficult to establish on a clinical and practical point of view and can vary in severity, there was uncertainty about the sarcopenia status of the patients in the rest of the four included studies. Although no formal sarcopenia assessment was mentioned in these studies, they reported outcomes of deprescribing on sarcopenia parameters [40–42, 45].

### Presence of polypharmacy

Three studies had inclusion criteria relating to polypharmacy, defined as taking 4 or more medicines or at least one anticholinergic or sedative medicine [45], taking 5 or more medicines [44] or taking 6 or more [43], respectively. The other three studies focused on the antihypertensive drug class in a frailty clinic [42] and comprehensive geriatric assessment (CGA) clinic setting [40] and benzodiazepine and z-drug withdrawal study (hereafter referred to as benzodiazepine withdrawal) in primary care [41]. The average number of medications at baseline across studies ranged from 4 to 8.

### Deprescribing interventions

In the studies that included sarcopenic patients who had stroke [43, 44], medications were reviewed by a hospital pharmacist on admission and potentially inappropriate medications were identified using the American Geriatrics Society 2019 Beers criteria [46]. This was in conjunction with physical therapy (up to 3 h per day) and nutrition management individualised to physical ability. The presence of deprescribing was defined as whether number of medications did or did not decrease between admission and discharge. There was a mean decrease of two drugs in the deprescribing group and a mean increase of one drug in the non-deprescribing group in both analyses [43, 44].

In the study based in the CGA clinic, patients received geriatrician-led medicines optimisation based on the CGA approach and 2015 Beers criteria [40]. Analysis was completed on the deprescribing group only before and after optimisation, the mean drug number decreased from 5.11 ± 3.34 to 4.76 ± 2.72 in the 6-month follow-up (*p* < 0.001). In the study on patients taking antihypertensives [42], these medicines were identified using Essence of Japanese Society of Hypertension Guidelines and deprescribing was defined as discontinuation of any of these medications. No details were provided on how and why antihypertensive medications were discontinued.

The single multicentre open-label parallel RCT in aged-care facilities [45] utilised a pharmacist-led intervention using validated tools to detect signs and symptoms of medicine-induced deterioration which occurred every 8 weeks over 12 months. This was compared to a control group receiving usual care which could involve one annual medication management review. Of the 309 recommendations made, 53% were to decrease dosage or cease use. At the level of the individual, pharmacists made recommendations to reduce medicine use for 61% of the population. No significant difference was observed for the rate of adverse events between arms (intervention rate per person per month 0.23 (± 0.32) versus 0.2 (± 0.36) in the comparison group, estimated rate ratio 1.12, 95%CI: 0.78–1.61, *p* = 0.55).

In the primary care study on withdrawal of benzodiazepines, individual physician-directed withdrawal of temazepam, zopiclone and zolpidem was performed gradually over a one-month period and participants were followed up to six months [41]. Withdrawal outcome and persistence were determined by plasma benzodiazepine-determinations at baseline and at one month (“short-term withdrawers”, *n* = 69) and by interviews at six months (“long-term withdrawers”, *n* = 34). It is noteworthy that even many of the non-withdrawers (both men and women) had also reduced their benzodiazepine use. For example, in the long-term non-withdrawer group (*n* = 55), 37 were using benzodiazepines once a week or less frequently, 7 were using a hypnotic 2–6 times a week, and 11 were daily users of benzodiazepines six months after starting the withdrawal.

## Deprescribing outcomes

### Primary outcomes: sarcopenia parameters

#### Muscle strength

Three studies included outcomes on handgrip strength (HG) measured using hand dynamometer and reported either some improvements or preservation of HG. In the primary care study of benzodiazepines withdrawal, within three weeks after initiating withdrawal, handgrip strength improved significantly (*p* ≤ 0.005) compared to baseline values [41]. Among women, long-term withdrawers improved their handgrip strength both when compared to their baseline values (*p* = 0.001) or to non-withdrawers (*p* = 0.004). In men, improvement of handgrip strength from baseline was not significantly better in withdrawers than in non-withdrawers. However, men did improve their handgrip strength values compared to baseline (*p* = 0.002). The multi-centre RCT in aged-care facilities observed a small increase of 0.53 kg (± 4.53) in the mean HG in the intervention group between baseline and at 12 months whereas mean HG decreased by 0.15 kg (± 4.07) in the control group [45]. However, there was no significant difference in grip strength between the intervention and control groups at 12 month follow up (modelled estimate 95%CI, 0.47 kg (− 0.71–1.66, *p* = 0.433). Point estimates favoured the intervention arm at 12 months for grip strength. However, not knowing the differences in deprescribing rates between intervention and control group make it difficult to associate any changes between groups in sarcopenia parameters with deprescribing. Among sarcopenic patients with stroke, the change in the number of medications was not statistically significantly associated with HG (β = 0.018, *p* = 0.768) at discharge [43].

#### Skeletal muscle mass or quality

Two studies reported outcomes of deprescribing on skeletal muscle mass Index (SMI). Using dual-energy X-ray absorptiometry and bioelectrical impedance analysis, selecting the lower value, SMI was significantly higher in the antihypertensive discontinuation group at baseline compared to the continuation group [42]. Within group analysis showed no changes in either group between baseline and 1-year follow up (discontinuation group 7.2 kg/m^2^ ± 1.7 vs. 6.9 kg/m^2^ ± 1.1, *p* = 0.24; continuation group 6.2 kg/m^2^ ± 1.0 vs. 6.1 kg/m^2^ ± 1.1, *p* = 0.15) [42]. Among sarcopenic patients with stroke, the change in the number of medications was not statistically significantly associated with SMI measured using BIA (β = 0.083, *p* = 0.265) at discharge [43].

#### Physical performance and function

Outcomes reported included gait speed (two studies) [40, 42], balance (two studies) [41, 42], Timed Up and Go (TUG) test (one study) [40], and Functional Independence Measure (FIM) (one study) [44].

##### Gait speed

two studies reported either preservation or improvement in gait speed. In the CGA clinic study [40], gait speed was preserved during the 6 months follow up (0.93 m/s ± 0.38 at baseline vs. 0.92 m/s ± 0.35 at follow up, *p* = 0.162). The study on antihypertensive discontinuation showed a statistically significant improvement in the Short Physical Performance Battery (SPPB) gait speed score (better performance) in both groups between baseline and 1-year follow up, (discontinuation group 2.9 m/s ± 1.1 vs. 3.4 ± 0.6, *p* < 0.05; continuation group 3.2 m/s ± 0.9 vs. 3.5 m/s ± 0.8, *p* < 0.01) [42], with no between group analysis conducted.

##### Balance

two studies reported potential improvements in balance following deprescribing of antihypertensive drugs and benzodiazepines. In the antihypertensive discontinuation study [42], the discontinuation group had a statistically significant increase in the SPPB balance score (*p* < 0.05), and total SPPB score (*p* < 0.05) between baseline and 1-year follow up. For the continuation group SPPB balance score decreased (*p* < 0.05), at the 1-year follow-up. No between groups comparison was reported. In the benzodiazepine’s withdrawal study, balance using the Short Berg’s Balance Scale (BBS-9 score) improved starting from the first week after withdrawal initiation. There was, however, only a borderline difference (*p* = 0.054) in balance improvement between the long-term withdrawers and long-term non-withdrawers.

##### TUG

There was no significant difference in TUG between baseline (14.79s ± 10.86) and 6-month follow up (13.78s ± 8.27) (*p* = 0.461) after geriatrician-led medication optimisation in the CGA clinic-based study [40].

Physical function: one study among people with sarcopenia after stroke measured Functional Independence Measure (FIM) scores for physical function (FIM-motor) and reported deprescribing was independently associated with better FIMmotor at discharge (β = 0.137; *p* = 0.017) and home discharge (odds ratio, 1.393; *p* = 0.002) [44].

#### Secondary outcomes: nutrition and energy intake (4 studies)

Among patients with sarcopenia following stroke [43], using multivariate linear regression, the decrease in the number of medications was independently associated with energy intake (β =-0.237, *p* = 0.009) and protein intake (β=-0.242, *p* = 0.047) at discharge [43]. In the study based at the CGA clinic [40], Mini-Nutritional Assessment (MNA) score significantly improved at the end of 6 months in the patients whose total number of drugs decreased (12.13 ± 2.23 at baseline vs. 12.69 ± 2.03 at 6-month follow up; p *<* 0.001). In the study on antihypertensive discontinuation, no significant changes in BMI or weight loss of 2 kg were observed between baseline and 1-year follow up in either group or between groups [42]. Whereas the RCT in aged-care facilities found a small mean weight loss in the intervention group and weight gain in the control group, representing a significant difference at 12 months (mean difference: 1.34 kg, 95%CI: −2.60, − 0.09; *p* = 0.035).

## Discussion

This is the first review to summarise the paucity of evidence on the effects of deprescribing on sarcopenia parameters. Amongst a low number of studies, across different settings and cohorts, and with heterogenous outcome measures, the results suggest there is no significant change in muscle mass with deprescribing. However, we found potentially some positive signs for improvements in grip strength and nutrition following deprescribing. Results in relation to physical function and performance showed no change or improvements based on the outcome measures used. For example, no significant changes in activity of daily living, Timed Up and Go and gait speed following deprescribing were observed. Whereas positive trends in balance were observed after deprescribing antihypertensives and benzodiazepines which could potentially be explained by reducing potential side effects of these drugs, rather than reversing sarcopenia. Given the heterogeneity in study designs and quality of included studies, meaningful conclusions are difficult to draw, and the review highlights a pressing need for further research in this area.

Several systematic reviews have synthesised the evidence on outcomes of deprescribing interventions among older people in general [47, 48], or defined by setting including care homes [49, 50], primary care and community [51, 52] and hospitals [53]. These reviews reported that deprescribing is feasible, well tolerated, safe, and generally effective in reducing the number of inappropriate prescriptions. This review adds more to the existing and expanding evidence of the effects of deprescribing in older populations by focusing on the muscle strength, mass/quality and function. In 2020, Aubert et al. reviewed the outcome measures used in 93 deprescribing intervention studies and reported that 97% used at least one measure related to appropriate prescribing, and only 34% used health and patient-reported measures [54]. The impact of deprescribing on some geriatric syndromes such as frailty and sarcopenia have rarely been reported [27]. Malnutrition, frailty, and sarcopenia often overlap and co-exist in older people, which suggests a common pathogenic mechanism [55]. A systematic review of the evidence of deprescribing among older people living with frailty concluded that stopping medications was feasible, safe, and could have a positive impact on depression and function within 6 months follow up [29]. Similarly, this review identified possible improvements in functional and nutritional status associated with deprescribing [56]. It is, however, important to note that deprescribing in the studies included in this review was not associated with deterioration in sarcopenia parameters adding to the evidence that deprescribing inappropriate medications is safe to undertake with potential cost savings. Effectively reducing medication burden through deprescribing with no change in clinical outcomes has been viewed positively in the literature [27].

The variation in the results of deprescribing on sarcopenia-related outcomes in the review may also relate to variation in the study design (majority were observational studies), sample size, quality of the included studies, variation in outcome measures and follow-up periods, and inclusion of both people with or without sarcopenia. Only two out of the six articles included older patients with confirmed sarcopenia diagnosis and showed that deprescribing can improve energy and protein intake but did not demonstrate any improvements in muscle mass or grip strength. Lack of positive results should be interpreted carefully because participants in these cohorts were post-stroke patients which might affect their function and muscle strength and the follow-up period was potentially too short (107 days) to show any significant differences.

Most of the studies included in this review have focused on any reduction in the number of medications to define their deprescribing group, with the exception of two studies that focused on specific drug classes (antihypertensives and benzodiazepines). This led to substantial heterogeneity in the types of medications stopped, many of which might be expected to have no impact on sarcopenia parameters. Indeed, the two studies that focused on antihypertensive discontinuation and benzodiazepines withdrawal showed positive outcomes on improving grip strength and balance. However, these studies did not include participants who had been diagnosed with sarcopenia, who may have different responses to deprescribing. This is key as future research could consider focusing on key drug classes likely to influence sarcopenia parameters through defined pathological mechanisms. It is also important to have consensus regarding the aim of future interventions, as deprescription of certain drugs classes may have a different role in prevention of sarcopenia versus improvement in functional parameters among those with sarcopenia. Future research may also investigate practical aspects of detecting changes in sarcopenia measures as a result in deprescribing. For example, which outcome/scale is most valid and reliable to use and when outcomes are best measured following deprescribing (i.e., if deprescribing does impact sarcopenia, when is a clinically meaningful change apparent and is the change durable over time? ). It is also important to understand whether deprescribing should be targeting specific medications that are likely to worsen sarcopenia to demonstrate any effectiveness. Deprescribing interventions were generic and did not target specific medication classes, apart from two studies targeted antihypertensives and benzodiazepines, and thus the observed no effects on function outcomes may reflect the lack of specificity.

A recent systematic review identified seven studies which demonstrated an association between polypharmacy and malnutrition regardless of the instrument or criterion used to define risk of malnutrition or polypharmacy [37]. Our review suggests potential improvements in nutritional parameters (intake and MNA score), which is interesting, since the mainstay of treatment for sarcopenia includes nutritional intervention. This finding may be clinically important, as it implies deprescribing inappropriate polypharmacy in patients with sarcopenia may promote improvement in nutritional status. Careful and individualized approaches to reducing unnecessary medications can lead to significant improvements in muscle health and overall physical performance. It is also important for design of future research, as nutritional outcomes should be included within outcomes measures, given a potential mediating effect on the relationship between deprescribing and sarcopenia parameters.

### Strength and limitations

Strengths of this study are its conduct in line with recommended best practices, including preregistration of the protocol, and the broad search strategy it used, developed with a senior librarian. A limitation was that the small number and heterogeneity of included studies precluded a quantitative synthesis. Studies included were mainly observational, and in some cases only reporting within group changes among those with deprescribing over time, without any comparison to control groups. In addition, only two of the included studies reported the outcomes of deprescribing among people who had confirmed diagnosis of sarcopenia. Due to the limited data and low number of included studies, the quality of included studies was not used our interpretation of the findings. To inform guidelines and recommendations for sarcopenia management, future research should focus on adequately powered high quality clinical trials, recruiting representative populations, to provide evidence on the added value of including deprescribing as part of patient’s treatment plan.

## Conclusion

There is a paucity of research about the impact of deprescribing inappropriate polypharmacy on sarcopenia parameters. The review highlights no significant changes in muscle mass, strength, and unclear effects on function with deprescribing, with some improvements in nutrition were observed. However, these findings come from a very small number of studies in heterogeneous populations and settings. There was no deterioration of sarcopenia parameters following deprescribing, which may suggest that it is worth implementing in this population due to the potential adverse events of medication and associated medication costs. More high-quality research is needed to understand the evidence for deprescribing among people with sarcopenia. To build the evidence in this important area, future deprescribing studies should consider including muscle-related outcomes (strength, mass/quality and function) in their research design. Furthermore, it is imperative to understand whether deprescribing certain medications known to worsen muscle health can improve sarcopenia parameters.

## Electronic supplementary material

Below is the link to the electronic supplementary material.

Supplementary Material 1

## Data Availability

All data available are included in the manuscript.
