# Supplementary material for: The evidence and impact of deprescribing on sarcopenia parameters: a systematic review
Source: BMC Geriatr. 2025 Mar 7;25:158. doi: 10.1186/s12877-025-05819-7 (PMC11887267; doi:10.1186/s12877-025-05819-7)
Supplement: Supplementary file 1 — Supplementary Material 1 [file 12877_2025_5819_MOESM1_ESM.docx]

**Cochrane library (Rayyan- RIS export citation 0-2)**

#1 deprescri*

#2 drug taper*

#3 inappropriate prescri*

#4 polypharmacy

#5 medication review

#6 medication reconcilliation

#7 drug substitution

#8 #1 or #2 or #3 or #4 or #5 or #6 or #7

#9 MeSH descriptor: [Muscle, Skeletal] explode all trees

#10 muscle weakness

#11 MeSH descriptor: [Muscular Atrophy] explode all trees

#12 MeSH descriptor: [Muscle Strength] explode all trees

#13 MeSH descriptor: [Gait] explode all trees

#14 #9 or #10 or #11 or #12 or #13

#15 #8 and #14 1022

**Embase Classic+Embase <1947 to 2023 June 28> via OVID (Rayyan- RIS 27-29 file upload)**

1 deprescri*.mp. 3122

2 drug taper*.mp. 192

3 inappropriate prescri*.mp. 7709

4 polypharmacy.mp. 28774

5 medication review.mp. 4082

6 medication reconciliation.mp. 4442

7 drug substitution.mp. 53084

8 1 or 2 or 3 or 4 or 5 or 6 or 7

9 exp muscle, skeletal/ 455511

10 muscle weakness.mp. 69897

11 exp muscular atrophy/ 62443

12 exp muscle strength/ 84223

13 exp gait/ 71107

14 9 or 10 or 11 or 12 or 13 647552

15 8 and 14 2021

**Ovid MEDLINE(R) ALL <1946 to June 28, 2023> via OVID (Rayyan- RIS 26 file upload)**

1 deprescri*.mp. 1996

2 drug taper*.mp. 505

3 inappropriate prescri*.mp. 6190

4 polypharmacy.mp. 13640

5 medication review*.mp. 2404

6 medication reconciliation.mp. 2635

7 drug substitution.mp. 4821

8 1 or 2 or 3 or 4 or 5 or 6 or 7 28433

9 exp Muscle, Skeletal/ 302637

10 Muscle Weakness.mp. 24668

11 exp Muscular Atrophy/ 21605

12 exp Muscle Strength/ 44677

13 exp Gait/ 36970

14 9 or 10 or 11 or 12 or 13 391700

15 8 and 14 177

**CINAHL via EBSCO (Rayyan- RIS 073fo49a file upload)**

| S16 | S8 AND S15 | 390 |
| --- | --- | --- |
| S15 | S9 or S10 or S11 or S12 or S13 or S14 |  |
| S14 | gait analysis |  |
| S13 | (MH “Gait+”) |  |
| S12 | (MH "Muscle Strength+") |  |
| S11 | (MH "Muscle, Skeletal+") |  |
| S10 | (MH "Muscular Atrophy+") |  |
| S9 | muscle weakness |  |
| S8 | S1 or S2 or S3 or S4 or S5 or S6 or S7 |  |
| S7 | drug substitution |  |
| S6 | medication reconciliation |  |
| S5 | medication review |  |
| S4 | (MH "polypharmacy+") |  |
| S3 | inappropriate prescri* |  |
| S2 | (MH "drug tapering") |  |
| S1 | deprescri* |  |
